# Gastrointestinal symptoms in the ICU: a comparison of primary and secondary acute gastrointestinal injury

**DOI:** 10.1186/cc12339

**Published:** 2013-03-19

**Authors:** A Reintam Blaser, J Starkopf

**Affiliations:** 1 University of Tartu, Tartu University Hospital, Tartu, Estonia

## Introduction

We aimed to clarify the differences between primary and secondary acute GI injury.

## Methods

A total of 2,690 consecutive adult patients were retrospectively studied during their first week in the ICU. Pathology in the GI system or laparotomy defined the primary GI insult. If GI symptoms developed without primary GI insult it was considered secondary GI injury. Absent bowel sounds (BS), vomiting/regurgitation, diarrhoea, bowel distension, GI bleeding, and high gastric residuals (GRV >1,000 ml/24 hours) were recorded daily.

## Results

**Figure 1:**
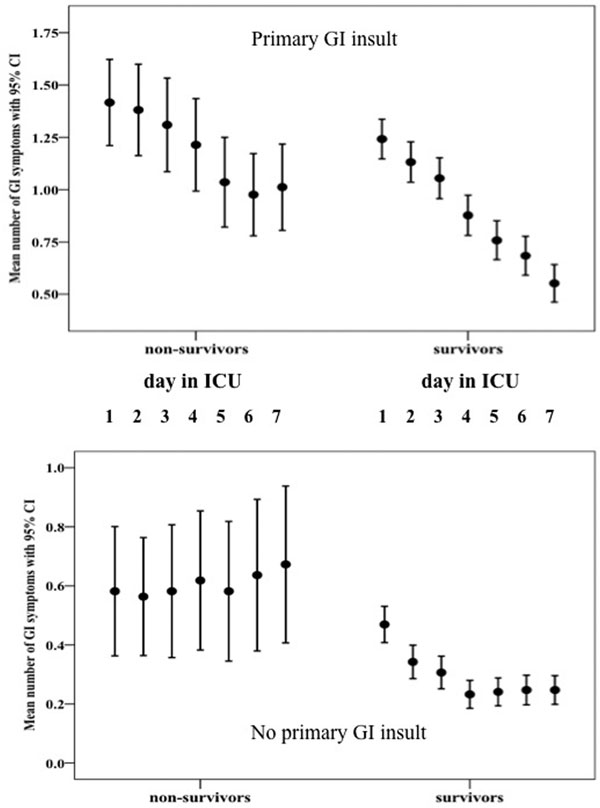
**Daily dynamics of the number of GI symptoms**. *P <*0.001 between survivors and nonsurvivors in all time points.

In total, 2,690 patients (60.4% male), median age 59 years (range 16 to 92), were studied. Eighty-four per cent of them were ventilated, 72% received vasopressor/inotrope. Median (IQR) APACHE II score was 14 (9 to 21) and SOFA on the first day was 6 (3 to 10). A total 35.5% had primary GI pathology. During the first week 34% of patients had absent BS, 38% vomiting/regurgitation, 9% diarrhoea, 7% bowel distension, 6% high GRV and 5% GI bleeding. All symptoms except diarrhoea occurred more often (<0.001) in patients with primary GI insult. Eighty-five per cent of patients with primary GI insult versus 46% without developed at least one GI symptom. The incidence of GI symptoms was significantly higher in nonsurvivors. ICU mortality was lower in patients with primary than secondary GI injury (43.6% vs. 61.2%, *P *= 0.046). Nonsurvivors without primary GI insult developed GI symptoms later (Figure 1).

## Conclusion

Primary and secondary acute GI injury have different incidence, dynamics and outcome.

